# Determinants of COVID-19 Vaccine Acceptance Among the General Adult Population in Saudi Arabia Based on the Health Belief Model: A Web-Based Cross-Sectional Study

**DOI:** 10.7759/cureus.28326

**Published:** 2022-08-23

**Authors:** Ezzuddin A Okmi, Emad Almohammadi, Olfat Alaamri, Rasha Alfawaz, Naif Alomari, Marwah Abdulaziz Saleh Alnughaymishi, Sulaiman Alsuwailem, Naseem J Moafa

**Affiliations:** 1 Department of Communicable Disease Prevention and Control, Saudi Public Health Authority, Riyadh, SAU; 2 Department of Health Promotion, Saudi Public Health Authority, Riyadh, SAU; 3 Department of Family of Medicine, King Saud Medical City, Riyadh, SAU; 4 Dentistry, Jazan University, Riyadh, SAU

**Keywords:** the kingdom of saudi arabia, health belief model, hesitancy, acceptance, vaccine, covid-19

## Abstract

Background

Many studies have been conducted worldwide and in the Kingdom of Saudi Arabia (KSA) during the current coronavirus disease 2019 (COVID-19) pandemic to assess the factors affecting COVID-19 vaccine acceptance. However, only some of these studies have adopted the Health Belief Model (HBM). This study aimed to assess the demographic characteristics and socio-psychological variables affecting the willingness to receive the COVID-19 vaccine among the general adult population in the KSA using the basic elements of the HBM.

Methods

A cross-sectional survey-based study was conducted. A Google Form questionnaire comprising 30 questions was distributed electronically using social media platforms. A univariate analysis using chi-square testing identified candidate variables for the multivariate logistic regression at a p-value of <.05 at 95% confidence interval (CI) set as a cut-off point. Multivariate logistic regression analysis was used to determine the association between multiple predictor variables and the dichotomized COVID-19 vaccine acceptance variable.

Results

A total of 1939 individuals participated in the current study. More than 73% were willing to take the vaccine, while the rest were either not willing (14.6%) or not sure (12.1%). The results showed that men were 1.29 times more likely to receive the COVID-19 vaccine than women (odds ratio, or OR = 1.29, 95% CI = 1.01-1.64, p = .04); those who were or had been a healthcare worker (HCW) were 1.43 times more likely to receive the COVID-19 vaccine compared with those who had never been a HCW (OR = 1.43, 95% CI = 1.10-1.87, p = .01). We found that perceiving the risk of contracting COVID-19 (OR = 2.86, 95% CI = 1.47-5.55, p = .00) and perceiving the severity of the disease (OR = 2.07, 95% CI = 1.08-3.96, p = .03) were positively associated with the willingness to receive the vaccine. Perceived barriers such as ineffectiveness of the vaccine (OR = 0.28, 95% CI = 0.18-0.44, p < .001), or believing the vaccine is just a media advertisement (OR = 0.56, 95% CI = 0.35-0.87, p = .01) were negative predictors of acceptance of the vaccine. Moreover, perceiving the benefits, such as life going back to normal (OR = 2.28, 95% CI = 1.37-3.77, p = .00) and recognizing the importance of the annual flu vaccine (OR = 3.43, 95% CI = 2.29-5.14, p < .001), were found to be positive predictors of acceptance of the vaccine. Finally, we also found that cues to action were positively associated with vaccine acceptance, that is, participants who were encouraged by their doctors (OR = 1.75, 95% CI = 1.17-2.60, p = .01), and family members or friends (OR = 2.89, 95% CI = 1.94-4.32, p* *< .001) were more willing to receive the COVID-19 vaccine than those who were not.

Conclusions

The current study provides valuable insights into the determinants of vaccine acceptance and hesitancy based on the HBM from a cognitive perspective. This could be useful in helping the government establish public health programs aimed at addressing barriers and false beliefs among the adult population, which could enhance the public’s willingness to receive COVID-19 vaccines and, ultimately, accelerate achieving herd immunity.

## Introduction

In December 2019, cases of fever and respiratory symptoms in China were brought about by an unknown pathogen, which was linked to the seafood wholesale market in Wuhan, China. The pathogen was eventually identified as a new coronavirus named as severe acute respiratory syndrome coronavirus 2 (SARS-CoV-2). Eventually, this clinical condition was named as coronavirus disease 2019 (COVID-19) by the World Health Organization (WHO) [1,2]. After an increase in the number of cases and thousands of deaths in China, the virus spread to several countries worldwide. Subsequently, the WHO declared COVID-19 as a pandemic due to the widespread infection and an increase in the daily number of new confirmed cases [3].

To mitigate the spread of SARS-CoV-2 infection and to minimize the impact on health facilities, the Kingdom of Saudi Arabia (KSA) and almost all countries implemented different preventive and control measures, such as complete lockdowns; shutting down government and private sector facilities such as schools, restaurants, and markets; and making the use of face masks and social distancing mandatory [4,5]. However, these measures, in addition to achieving herd immunity through recovery from natural infection, were ineffective per se in both controlling the spread of COVID-19 infection and in ending the pandemic. Therefore, there was an urgent need to develop a vaccine against COVID-19 because immunization is historically known as a long-term strategy for the prevention of most infectious diseases [4,6].

However, conspiracy theories about COVID-19 vaccines and misinformation from non-credible sources are some of the most important barriers that may highly affect vaccine acceptance among the general adult population and can cause vaccine hesitancy [7]. The WHO defines vaccine hesitancy as a behavior affected by many factors, such as not trusting the vaccine itself or the provider or companies that produce such vaccines, and not knowing the value of or perceiving the need for vaccines [8]. There are different degrees of vaccine hesitancy; some people may delay receiving the vaccine and some may accept some vaccines and refuse others. Moreover, some refuse to receive the vaccine altogether. Some individuals accept all vaccines but still harbor concerns about their safety and efficacy [8,9]. Thus, to increase rates of COVID-19 vaccination acceptance, stakeholders in public health will need to respond with new frameworks targeted at beliefs and perceptions about the COVID-19 vaccine.

The Health Belief Model (HBM), which was initially developed in the 1950s, is considered one of the most widely used conceptual frameworks in health behavior research. The HBM can explain the reasons for refusal to receive preventive health interventions such as vaccination [10-12]. According to the HBM, individuals are more likely to receive a vaccine if they think they are susceptible to the disease targeted by the vaccine, think the disease targeted by the vaccine is a serious condition, believe vaccination against that disease will reduce their susceptibility to contracting it or make symptoms less severe, and believe the benefits of vaccination outweigh its disadvantages or barriers [12]. The HBM has been used in many studies investigating the vaccination acceptance rate for numerous conditions [11,12].

In the current COVID-19 pandemic, many studies have been conducted regionally in the KSA and worldwide to determine the factors affecting COVID-19 vaccine acceptance [13-26]. However, only some of these studies have adopted the HBM [27-30]. Thus, our study aimed to assess the demographic characteristics and socio-psychological variables affecting the intention to receive COVID-19 vaccination among the general adult population in the KSA based on the basic elements of the HBM.

## Materials and methods

Study design

A cross-sectional study was conducted from June 9 to July 23, 2021. A Google Form questionnaire comprising 30 questions written in both Arabic and English was distributed electronically using social media platforms, including WhatsApp, Twitter, and Instagram. We used snowball sampling to select prospective participants across all the principal regions of the KSA (central, eastern, western, northern, and southern regions). The participants were asked to share the link to the questionnaire with their contacts. Given the risk of spreading COVID-19, an online survey was reasonable to avoid physical contact.

Sample size calculation and participants

The sample size calculated using Raosoft (Raosoft Inc., Seattle, WA) was 385 for each region of the KSA, assuming a 50% response rate, 95% confidence interval (CI), 1.96 Z score, and 5% margin of error. Therefore, the total sample included 1925 (5×385) participants, as we geographically divided the country into five main regions.

The inclusion criteria were as follows: 18 years or older, currently residing in the KSA, had not received the COVID-19 vaccine, and were willing to complete the questionnaire. There were no exclusion criteria; those respondents who did not meet these criteria were excluded from the study. Of the 2289 who responded, 350 did not meet the criteria for inclusion. There were 22 participants who were under 18 years of age, 53 who did not currently reside in the KSA, 261 had already been vaccinated against COVID-19, and 14 respondents were unwilling to complete the questionnaire. The final sample consisted of 1939 participants.

Data collection

The initial draft of the questionnaire was developed in English and then translated to Arabic based on the previous literature exploring vaccination acceptance [11,12]. Then, it was reviewed by two expert researchers with medical backgrounds and subsequently validated.

In order to pre-test the questionnaire and evaluate content validity, we invited 100 individuals using WhatsApp to participate in completing the questionnaire. Sixty-two of them responded, and we randomly chose five individuals to complete the online self-administered questionnaire. The five participants included one high school student, two with an undergraduate degree, and two with a postgraduate degree. After they completed the questionnaire, we conducted face-to-face interviews with each one of them to ensure that they had understood the questions in the manner in which they were intended. Based on the pre-test of the questionnaire, we further modified some questions and response options to ensure the clarity of the questionnaire.

The questionnaire included the following sections: socio-demographic characteristics, health status of the participants, COVID-19-related experience, willingness to receive a COVID-19 vaccine, and HBM elements related to COVID-19 vaccination. HBM elements included perceived susceptibility, perceived severity, perceived benefits of a COVID-19 vaccine, perceived barriers to receiving the vaccination, and cues to action.

To assess the participants’ characteristics, we asked questions about age, gender, nationality, level of education, occupation, monthly income in Saudi Riyal (SR), and area of residence. To investigate the health status of the participants, we included a question about the diagnosis of chronic diseases. To assess prior experience of the participants with COVID-19, we asked about the COVID-19 infection history of the participants, their friends, and their family members. The participants’ willingness to receive a COVID-19 vaccine was measured using a question with three response options (“yes,” “no,” or “not sure”).

To assess the constructs of the HBM, we asked one question to measure perceived susceptibility, one question to assess perceived severity, three questions to determine the perceived benefits of a COVID-19 vaccine, three questions to assess perceived barriers to receiving the vaccination, and two questions to assess cues to action. We used three response options for each question: agree, neutral, and disagree.

Ethical considerations

Approval from the Institutional Review Board of the Saudi Center for Disease Prevention and Control was obtained (SCDC-IRB-A015-2020). An informed consent statement was included in the initial page of the online questionnaire. All participants were informed that participating in the study was voluntary, and hence they were asked whether they agreed to participate before filling the online questionnaire. The participants were informed about the purpose of the study as well. The data for this study were kept confidential and were used for the study’s purpose only.

Statistical analysis

Descriptive analysis was used to describe the participants’ characteristics. Means and standard deviations were used for continuous variables, whereas frequencies and proportions were used for categorical variables. The dependent variable of interest was the willingness to receive the COVID-19 vaccine, which collected data in three categories (“yes,” “no,” or “not sure”). For the analyses, we added the answer “no” to the answer “not sure.” Then, the three responses were re-categorized into dichotomous variables “yes” and “no.”

A univariate analysis using chi-square testing identified candidate variables for the multivariate logistic regression at a p-value of <0.05 at 95% CI set as a cut-off point. Multivariate logistic regression analysis was used to determine the association between multiple predictor variables and the dichotomized COVID-19 vaccine acceptance variable. No backward selection was used, and only variables with a significance level of <.05 remained in the model. All data analyses were performed using IBM SPSS Statistics, version 28 (IBM Corp., Armonk, NY).

## Results

A total of 1939 individuals were included in the current study, of which 57.2% were men. The mean age was 39 years with a standard deviation of 11 years. Almost all (92.4%) of the participants were Saudi, and half (50.7%) had graduated from a university. Most (79.5%) were married, and about 30% of the participants had a monthly income of less than 5000 SR. Half (50.4%) of the participants were government employees, and more than one-third (38.8%) were working or had worked as healthcare workers (HCWs). Approximately 20% had chronic diseases, about 15% had had COVID-19, and about 67% reported that one of their family members or friends had been infected with COVID-19. Most participants (73.3%) were willing to take the vaccine, while 14.6% were not willing to do so, and 12.1% were not sure (Table 1).

**Table 1:** Participants’ characteristics (n=1939) COVID-19, coronavirus disease 2019; SR, Saudi Riyal

Variable	Frequency	%
Gender		
Male	1110	57.2
Female	829	42.8
Age (years)		
18–29	375	19.3
30–44	1005	51.8
45–59	479	24.7
60 or above	80	4.1
Mean	38.71	
SD	10.56	
Marital status		
Single	398	20.5
Married	1541	79.5
Nationality		
Non-Saudi	148	7.6
Saudi	1791	92.4
Level of education		
High school education or less	265	13.7
Diploma (a 2-year associate degree post-high school)	356	18.4
University graduates	984	50.7
Higher education (postgraduates)	334	17.2
Occupation		
Unemployed (retired, housewife)	457	23.6
Governmental sector employee	978	50.4
Private sector employee	279	14.4
Military sector employee	113	5.8
Student	112	5.8
Are you working/have you worked as a healthcare worker?		
No	1186	61.2
Yes	753	38.8
Monthly income (SR)		
<5000	582	30.0
5000–10,000	426	22.0
10,000–15,000	462	23.8
15,000–20,000	292	15.1
>20,000	177	9.1
Area of residence		
South	303	15.6
Central	811	41.8
North	148	7.6
West	367	18.9
East	310	16.0
Do you have any chronic diseases?		
No	1559	80.4
Yes	380	19.6
Have you ever had COVID-19?		
No	1647	84.9
Yes	292	15.1
Has any of your family members or friends ever been infected with COVID-19?		
No	642	33.1
Yes	1297	66.9
Are you willing to receive the vaccine?		
No	283	14.6
Yes	1422	73.3
Not sure	234	12.1

We conducted univariate analysis using chi-square testing to assess the association between the acceptance of the COVID-19 vaccine and participants’ characteristics (Table 2), and to determine the association between the acceptance of the COVID-19 vaccine and HBM factors (Table 3). Among all participants’ characteristics, there was a statistically significant association for gender (p < .001), level of education (p = .015), occupation (p = .005), being an HCW or being an HCW in the past (p < .001), and monthly income (p = .033). Moreover, there were statistically significant associations between the acceptance of the COVID-19 vaccine and all elements of the HBM (Table 3).

**Table 2:** Association between participants’ characteristics and willingness to receive the COVID-19 vaccination (n=1939), found using the chi-square test COVID-19, coronavirus disease 2019; SR, Saudi Riyal *p value is significant at <.05.

Variable	Are you willing to receive the vaccine?		Chi-square	p value
	No	Yes		
	%	%		
Age (years)			5.676	.128
18–29	116	259		
	22.4%	18.2%		
30–44	267	738		
	51.6%	51.9%		
45–59	116	363		
	22.4%	25.5%		
60 or above	18	62		
	3.5%	4.4%		
Gender			11.708	< .001*
Male	263	847		
	50.9%	59.6%		
Female	254	575		
	49.1%	40.4%		
Marital status			0.013	.911
Single	107	291		
	20.7%	20.5%		
Married	410	1131		
	79.3%	79.5%		
Nationality			3.349	.067
Non-Saudi	30	118		
	5.8%	8.3%		
Saudi	487	1304		
	94.2%	91.7%		
Level of education			10.484	.015*
High school education or less	73	192		
	14.1%	13.5%		
Diploma (a 2-year associate degree post-high school)	76	280		
	14.7%	19.7%		
University graduates	289	695		
	55.9%	48.9%		
Higher education (postgraduates)	79	255		
	15.3%	17.9%		
Occupation			14.795	.005*
Unemployed (retired, housewife)	140	317		
	27.1%	22.3%		
Governmental sector employee	230	748		
	44.5%	52.6%		
Private sector employee	71	208		
	13.7%	14.6%		
Military sector employee	37	76		
	7.2%	5.3%		
Student	39	73		
	7.5%	5.1%		
Are you working/have you worked as a healthcare worker?			20.315	< .001*
No	359	827		
	69.4%	58.2%		
Yes	158	595		
	30.6%	41.8%		
Monthly income (SR)			10.485	.033*
<5000	404	582		
	28.4%	30.0%		
5000–10,000	313	426		
	22.0%	22.0%		
10,000–15,000	338	462		
	23.8%	23.8%		
15,000–20,000	227	292		
	16.0%	15.1%		
>20,000	140	177		
	9.8%	9.1%		
Area of residence			8.356	.079
South	79	224		
	15.3%	15.8%		
Central	228	583		
	44.1%	41.0%		
North	25	123		
	4.8%	8.6%		
West	99	268		
	19.1%	18.8%		
East	86	224		
	16.6%	15.8%		
Do you have any chronic diseases?			0.185	.668
No	419	1140		
	81.0%	80.2%		
Yes	98	282		
	19.0%	19.8%		
Have you ever had COVID-19?			1.617	.203
No	448	1199		
	86.7%	84.3%		
Yes	69	223		
	13.3%	15.7%		
Has any of your family members or friends ever been infected with COVID-19?			1.958	.162
No	184	458		
	35.6%	32.2%		
Yes	333	964		
	64.4%	67.8%		

**Table 3:** Association between HBM-related elements and willingness to receive COVID-19 vaccination (n=1939), found using the chi-square test COVID-19, coronavirus disease 2019; HBM, Health Belief Model *p value is significant at <.05.

Variable		Are you willing to receive the vaccine?		Chi-square	p value
		No	Yes		
		%	%		
Perceived susceptibility					
Am I at high risk of contracting COVID-19 currently or in the future?	Disagree	329	227	513.860	< .001*
		63.6%	16.0%		
	Agree	52	877		
		10.1%	61.7%		
	Neutral	136	318		
		26.3%	22.4%		
Perceived severity					
Will I be very sick or die if I get COVID-19?	Disagree	347	239	529.560	< .001*
		67.1%	16.8%		
	Agree	55	894		
		10.6%	62.9%		
	Neutral	115	289		
		22.2%	20.3%		
Perceived benefits					
Getting the flu vaccine annually is very important for me	Disagree	358	482	212.027	< .001*
		69.2%	33.9%		
	Agree	48	505		
		9.3%	35.5%		
	Neutral	111	435		
		21.5%	30.6%		
Getting vaccinated is the best way to avoid being severely infected with COVID-19 compared to other preventive measures (hand washing, wearing a mask, and social distancing)	Disagree	186	156	378.032	< .001*
		36.0%	11.0%		
	Agree	104	981		
		20.1%	69.0%		
	Neutral	227	285		
		43.9%	20.0%		
Vaccinating most of the community with the COVID-19 vaccine will control the virus and life will get back to normal	Disagree	149	68	455.439	< .001*
		28.8%	4.8%		
	Agree	116	1044		
		22.4%	73.4%		
	Neutral	252	310		
		48.7%	21.8%		
Perceived barriers					
I am not planning to receive the COVID-19 vaccine because I am concerned about the vaccine’s side effects	Disagree	62	375	68.861	< .001*
		12.0%	26.4%		
	Agree	372	734		
		72.0%	51.6%		
	Neutral	83	313		
		16.1%	22.0%		
I am not planning to receive the vaccine because it is just an advertisement in the media	Disagree	137	1034	367.935	< .001*
		26.5%	72.7%		
	Agree	187	125		
		36.2%	8.8%		
	Neutral	193	263		
		37.3%	18.5%		
I am not planning to receive the vaccine because I don’t believe it’s efficient	Disagree	76	929	454.309	< .001*
		14.7%	65.3%		
	Agree	253	168		
		48.9%	11.8%		
	Neutral	188	325		
		36.4%	22.9%		
Cues to action					
I will probably be encouraged to receive the vaccine if one of my family members or friends has received it before	Disagree	250	188	424.401	< .001*
		48.4%	13.2%		
	Agree	98	991		
		19.0%	69.7%		
	Neutral	169	243		
		32.7%	17.1%		
My acceptance of getting vaccinated depends on my doctor’s recommendation	Disagree	209	207	218.552	< .001*
		40.4%	14.6%		
	Agree	156	933		
		30.2%	65.6%		
	Neutral	152	282		
		29.4%	19.8%		

Furthermore, we used multivariate logistic regression to adjust the model. Based on the findings, the adjusted odds ratio (aOR) confirmed that male gender and being a healthcare worker or having worked as a healthcare worker in the past were independent predictors of receiving the COVID-19 vaccine. Table 4 shows that men were 1.29 times more likely to receive the COVID-19 vaccine than women (aOR = 1.29, 95% CI = 1.01-1.64, p = .04), and those who were working or had worked as HCWs were 1.43 times more likely to receive the COVID-19 vaccine than those who had never been a healthcare worker (aOR = 1.43, 95% CI = 1.10-1.87, p = .01).

**Table 4:** Multivariate logistic regression model for the prediction of the willingness to receive the COVID-19 vaccine based on participants' characteristics (n=1939) COVID-19, coronavirus disease 2019; SR, Saudi Riyal; aOR, adjusted odds ratio; CI, confidence interval; r, reference *p value is significant at <.05.

Variable	aOR	95% CI		p value
		Lower	Upper	
Gender				
Female (r)	1			
Male	1.29	1.01	1.64	.04*
Level of education				
High school education or less (r)	1			
Diploma (a 2-year associate degree post-high school)	1.06	0.72	1.58	.77
University graduates	0.83	0.60	1.14	.25
Higher education (postgraduates)	0.91	0.60	1.38	.65
Occupation				
Unemployed (retired, housewife) (r)	1			
Governmental sector employee	1.00	0.72	1.38	.98
Private sector employee	1.13	0.79	1.62	.51
Military sector employee	0.70	0.42	1.14	.15
Student	0.79	0.50	1.25	.32
Are you working/have you worked as a healthcare worker?				
No (r)	1			
Yes	1.43	1.10	1.87	.01*
Monthly income (SR)				
<5000 (r)	1			
5000–10,000	0.99	0.72	1.34	.93
10,000–15,000	1.00	0.73	1.38	.98
15,000–20,000	1.24	0.85	1.80	.26
>20,000	1.37	0.87	2.16	.17

Moreover, the adjusted odds ratio confirmed the significant associations between the willingness to receive the COVID-19 vaccine and most items of the HBM elements (Table 5). Table 5 shows that participants who believed they were at high risk of COVID-19 infection currently or even in the future were 2.86 times more likely to accept the vaccine than those who did not believe so (aOR = 2.86, 95% CI = 1.47-5.55, p = .00). Participants who believed they would become very sick or die if they contracted COVID-19 were about two times more likely to receive the vaccine than those who did not believe so (aOR = 2.07, 95% CI = 1.08-3.96, p = .03). When considering the perceived benefits of the vaccine, we found that participants who considered receiving flu vaccines as highly important were 3.43 times more likely to accept COVID-19 vaccination than those who consider receiving flu vaccines as less important (aOR = 3.43, 95% CI = 2.29-5.14, p < .001). Participants who agreed that vaccinating most of the community will control the virus and bring the situation back to normal were about two times more likely to receive the vaccine than those who disagreed (aOR = 2.28, 95% CI = 1.37-3.77, p = .00). However, there was no statistically significant result for those who agreed that being vaccinated was the best measure to prevent severe COVID-19 infection compared to other measures of prevention (aOR = 1.11, 95% CI = 0.70-1.75, p = .66).

**Table 5:** Multivariate logistic regression model for the prediction of the willingness to receive the COVID-19 vaccine based on HBM elements (n=1939) COVID-19, coronavirus disease 2019; HBM, Health Belief Model; aOR, adjusted odds ratio; CI, confidence interval; r, reference *p value is significant at <.05.

Variable		aOR	95% CI		p value	
			Lower	Upper		
Perceived susceptibility						
Am I at high risk of getting COVID-19 currently or in the future?	Disagree (r)	1				
	Agree	2.86	1.47	5.55		.00*
	Neutral	1.22	0.72	2.06		.45
Perceived severity						
Will I be very sick or die if I get COVID-19?	Disagree (r)	1				
	Agree	2.07	1.08	3.96		.03*
	Neutral	1.53	0.89	2.63		.12
Perceived benefits						
Getting the flu vaccine annually is very important for me	Disagree (r)	1				
	Agree	3.43	2.29	5.14		< .001*
	Neutral	2.00	1.44	2.76		< .001*
Getting vaccinated is the best way to avoid getting severely infected with COVID-19 and its complications compared to other preventive measures (hand washing, wearing a mask, and social distancing)	Disagree (r)	1				
	Agree	1.11	0.70	1.75		.66
	Neutral	.90	0.60	1.34		.60
Vaccinating most of the community with the COVID-19 vaccine will control the virus and life will get back to normal	Disagree (r)	1				
	Agree	2.28	1.37	3.77		.00*
	Neutral	1.83	1.16	2.89		.01*
Perceived barriers						
I am not planning to receive the COVID-19 vaccine because I am concerned about the vaccine’s side effects	Disagree (r)	1				
	Agree	1.09	0.70	1.69		.71
	Neutral	1.26	0.74	2.13		.39
I am not planning to receive the vaccine because it is just an advertisement in the media	Disagree (r)	1				
	Agree	.56	0.35	0.87		.01*
	Neutral	.51	0.35	0.75		< .001*
I am not planning to receive the vaccine because I don’t believe it is efficient	Disagree (r)	1				
	Agree	.28	0.18	0.44		< .001*
	Neutral	.54	0.36	0.83		.00*
Cues to action						
My acceptance of getting vaccinated depends on my doctor’s recommendation	Disagree (r)	1				
	Agree	1.75	1.17	2.60		.01*
	Neutral	1.50	1.00	2.26		.05
I will probably be encouraged to take the vaccine if one of my family members or friends has taken it before	Disagree (r)	1				
	Agree	2.89	1.94	4.32		< .001*
	Neutral	1.29	0.87	1.92		.20

We investigated certain perceived barriers to being vaccinated, comparing those who agreed versus those who disagreed that these barriers prevented them from being vaccinated. The results indicated that those who disagreed that the vaccine is just an advertisement in the media were 1.79 times more accepting of the COVID-19 vaccine compared to those who agreed (aOR = 0.56, 95% CI = 0.35-0.87, p = .01), and those who disagreed that the COVID-19 vaccine is ineffective were 3.6 times more accepting of the vaccine compared with those who agreed (aOR = 0.28, 95% CI = 0.18-0.44, p < .001). The vaccine’s side effects were not perceived as a barrier. There was no statistically significant difference regarding COVID-19 vaccine acceptance between those who were concerned about side effects compared with those who were not concerned (aOR = 1.09, 95% CI = 0.70-1.69, p = .71).

Finally, the cues to action indicated that those who stated that their acceptance of the vaccine depended on their doctor’s recommendation were 1.75 times more accepting of the COVID-19 vaccine than those who disagreed that their acceptance of the vaccine depended on their doctor’s recommendation (aOR = 1.75, 95% CI = 1.17-2.60, p = .01). Additionally, those who agreed to take the vaccine if one of their family members or friends was vaccinated were 2.89 times more willing to receive the COVID-19 vaccine than those whose family members or friends were not vaccinated (aOR = 2.89, 95% CI = 1.94-4.32, p < .001).

## Discussion

Although there are a few studies on factors that influence COVID-19 vaccination acceptance among the general adult population in the KSA, studies on COVID-19 vaccine acceptance in the KSA using the HBM theory are scant [13-16]. Mahmud et al. conducted a study to predict the intention to receive the COVID-19 vaccine using the HBM in the KSA and found that older people and HCWs were more likely to receive the vaccine [27]. Moreover, the perception of vulnerability to and severity of COVID-19, and perceived benefits of the vaccine were positively associated with the vaccine uptake. However, in their study, two-thirds of the participants were from the central region, which could limit the generalization of the study to other regions. Therefore, in our study we included a larger sample size in each area using the HBM to explore the determinants of COVID-19 vaccine acceptance.

We gathered data using an online self-administered questionnaire and included men and women from all regions of the KSA who had not been vaccinated. The results indicated that of 1939 participants, 1426 (73.4%) were willing to receive the vaccine, while 283 (14.6%) answered “no” to being vaccinated and 234 (12%) answered “not sure” about receiving the vaccine. Furthermore, among the participants’ characteristics, gender and being a healthcare worker/having worked as a healthcare worker independently predicted who were willing to take the vaccine. Moreover, most items of the HBM elements were statistically significant predictors of acceptance of the COVID-19 vaccine.

We found that perceiving the risk of contracting COVID-19 and perceiving the severity of the disease were positively associated with the willingness to receive the vaccine. Perceived barriers such as ineffectiveness of the vaccine or believing the vaccine is just a media advertisement were negative predictors of acceptance of the vaccine. Moreover, perceiving the benefits, such as life going back to normal, and recognizing the importance of the annual flu vaccine, were found to be positive predictors of the acceptance of the vaccine. Finally, we also found that cues to action were positively associated with vaccine acceptance, that is, participants who were encouraged by their doctors, family members, or friends were more willing to receive the COVID-19 vaccine than those who were not.

In this study, the rate of acceptance of the COVID-19 vaccine was 73.4%. This rate resembles rates reported in previous studies conducted in countries such as China, USA, and many countries [20,21]. In our study, the acceptance rate was higher than the rates previously reported among the general adult population in the KSA [13-16]. Regionally, the highest rate was 64.7%, while the lowest rate was 48% [13,14]. One reason that could explain this observed high rate in our study is that our study was conducted during the period of the vaccination campaign, which might have alleviated worries among the population and increased intentions to be vaccinated. According to a systematic review of many studies in different countries, the rate in our study was within the global range as reported [17].

Gender was a significant predictor of COVID-19 vaccine acceptance, as reported in several studies [18,22,23]. In our study, men had higher odds of accepting the COVID-19 vaccine than women. This is similar to studies conducted in Kuwait and Hong Kong [22,23]. Moreover, it was stated in a systematic review that the majority of studies included showed men were more likely than women to receive the COVID-19 vaccine [17]. In contrast, in one study, it was reported that women were more willing to receive the COVID-19 vaccine [18]. This may be due to the variability in sample characteristics across studies, as some studies, such as a study conducted in Italy, included a university population [18]. This gender-based acceptance difference can be explained by the fact that women have specific concerns regarding the COVID-19 vaccines. For example, there is the false belief among women that vaccines may cause infertility, and women tend to be more fearful of adverse events and side effects of the COVID-19 vaccines [23].

Our study showed that being an HCW is linked with a high rate of COVID-19 vaccine acceptance. This result is congruent with the results of studies conducted previously [19,27]. This association is expected because HCWs are at high risk of being infected owing to their exposure to a high number of COVID-19 cases [17].

Regarding the HBM, our results are congruent with another study in the KSA. Mahmud et al. concluded that individuals who agreed on perceived susceptibility, perceived severity, perceived benefits, and cues to actions were more willing to receive the COVID-19 vaccine than those who disagreed. Conversely, people who disagreed on perceived barriers were more inclined to receive the COVID-19 vaccine [27].

Perceived risk and perceived severity of COVID-19 have been linked to increased vaccine acceptance [27,29,30]. Correspondingly, our study showed that participants who believed they were at high risk of contracting COVID-19 were about three times more likely to receive the vaccine than those who did not. Furthermore, participants who believed they would be very sick or die if they contracted COVID-19 were about two times more likely to accept the vaccine than those who did not believe so. However, Cai et al. found no significant association between perceived severity and uptake of the COVID-19 vaccine [28]. Furthermore, Cai et al. found opposing results to our study regarding perceived susceptibility. This discrepancy between our study and Cai et al.’s study could be explained by how different populations in different countries classify the risk of contracting the disease as mild or severe according to their level of knowledge.

In our study, participants who believed that the annual seasonal flu vaccine was highly important were more willing to take the COVID-19 vaccine than those who ascribed a low level of importance to such a vaccine. This is quite similar to what was reported in a systematic review about COVID-19 vaccine acceptance, which concluded that poor influenza vaccination history can result in vaccine hesitancy [17].

Doctors’ recommendations were also found to be a predictor of vaccine uptake in this study. This is similar to the study conducted in Kuwait that stated that high confidence in medical professionals is associated with an increased rate of vaccine uptake [26]. Furthermore, a study conducted in China confirmed the association between doctors’ support and vaccine uptake [25]. In addition, we found that participants who were encouraged by family members or friends were more inclined to take the vaccine than those who were not. This is congruent with a study conducted previously in Australia indicating that family support greatly increased vaccine acceptance [24].

Misinformation about the COVID-19 vaccine was reported as a reason for vaccine hesitancy [17]. In the current study, perceived barriers was negatively linked with vaccine acceptance. This resembles the findings of many previous studies assessing vaccine acceptance using the HBM [27-30].

Implications

Efforts to increase public willingness to receive COVID-19 vaccines are required to prevent spread of infection and establish herd immunity. Therefore, the current study provides valuable insights into the determinants of vaccine acceptance and hesitancy based on the HBM from the cognitive perspective, which could be useful for the decision-maker to establish public health programs, such as a health awareness programs or extensive educational programs through TV and social media addressing the barriers and false beliefs among the adult population in this study. Furthermore, this study has important implications for designing and implementing public health programs to enhance COVID-19 vaccine uptake. For example, health authorities must consider gender-based interventions, and public health intervention programs need to focus on increasing the perception of susceptibility to and severity of COVID-19 besides increasing the perceived benefits of vaccination while reducing the identified barriers.

Limitations

Our study has certain limitations that must be considered. First, because this is a cross-sectional study, causality cannot be established. Second, selection bias is a possible limitation as we used non-probability sampling, and we had no control over distributing the questionnaire using the random sampling method. Therefore, the data in this study might not accurately represent adults in Saudi Arabia. Third, for the purpose of dichotomous analysis, the “no” category was combined with the “not sure” category, and that may not be the actual representation of risk estimates in certain variables.

## Conclusions

Our research is one of the few studies carried out in the KSA employing the HBM to investigate the determinants of acceptance of the COVID-19 vaccine among the general adult population. We included a larger sample size from all regions of the country and found that men and HCWs were more likely to accept the vaccine. Moreover, we found that the elements of the HBM, such as perceived risk, perceived severity, perceived benefits, and cues to action were positive predictors of the acceptance of the COVID-19 vaccine, whereas perceived barriers were negatively linked to vaccine acceptance. Therefore, the findings of our study will be useful to decision-makers and health authorities, who must establish public health programs that aim to enhance the public’s willingness to receive COVID-19 vaccines and, ultimately, accelerate achieving herd immunity. We recommend that a prospective study with random probability sampling and face-to-face interviewing be conducted to confirm our findings.
